# Walking cadence (steps/min) and intensity in 21–40 year olds: CADENCE-adults

**DOI:** 10.1186/s12966-019-0769-6

**Published:** 2019-01-17

**Authors:** Catrine Tudor-Locke, Elroy J. Aguiar, Ho Han, Scott W. Ducharme, John M. Schuna, Tiago V. Barreira, Christopher C. Moore, Michael A. Busa, Jongil Lim, John R. Sirard, Stuart R. Chipkin, John Staudenmayer

**Affiliations:** 1 Department of Kinesiology, University of Massachusetts Amherst, 160A Totman Building, 30 Eastman Lane, Amherst, MA 01003 USA; 2 School of Community Health Sciences, Counseling and Counseling Psychology, Oklahoma State University, Stillwater, OK 74078 USA; 3 School of Biological and Population Health Sciences, Oregon State University, Corvallis, OR 97331 USA; 4 School of Education, Syracuse University, Syracuse, New York 13244 USA; 5 Institute for Applied Life Sciences, University of Massachusetts Amherst, Amherst, MA 01003 USA; 6 Department of Counseling, Health and Kinesiology, Texas A&M University - San Antonio, San Antonio, TX 78224 USA; 7 Department of Mathematics and Statistics, University of Massachusetts Amherst, Amherst, MA 01003 USA

**Keywords:** Physical activity, Pedometer, Accelerometer, Exercise

## Abstract

**Background:**

Previous studies have reported that walking cadence (steps/min) is associated with absolutely-defined intensity (metabolic equivalents; METs), such that cadence-based thresholds could serve as reasonable proxy values for ambulatory intensities.

**Purpose:**

To establish definitive heuristic (i.e., evidence-based, practical, rounded) thresholds linking cadence with absolutely-defined moderate (3 METs) and vigorous (6 METs) intensity.

**Methods:**

In this laboratory-based cross-sectional study, 76 healthy adults (10 men and 10 women representing each 5-year age-group category between 21 and 40 years, BMI = 24.8 ± 3.4 kg/m^2^) performed a series of 5-min treadmill bouts separated by 2-min rests. Bouts began at 0.5 mph and increased in 0.5 mph increments until participants: 1) chose to run, 2) achieved 75% of their predicted maximum heart rate, or 3) reported a Borg rating of perceived exertion > 13. Cadence was hand-tallied, and intensity (METs) was measured using a portable indirect calorimeter. Optimal cadence thresholds for moderate and vigorous ambulatory intensities were identified using a segmented regression model with random coefficients, as well as Receiver Operating Characteristic (ROC) models. Positive predictive values (PPV) of candidate heuristic thresholds were assessed to determine final heuristic values.

**Results:**

Optimal cadence thresholds for 3 METs and 6 METs were 102 and 129 steps/min, respectively, using the regression model, and 96 and 120 steps/min, respectively, using ROC models. Heuristic values were set at 100 steps/min (PPV of 91.4%), and 130 steps/min (PPV of 70.7%), respectively.

**Conclusions:**

Cadence thresholds of 100 and 130 steps/min can serve as reasonable heuristic thresholds representative of absolutely-defined moderate and vigorous ambulatory intensity, respectively, in 21–40 year olds. These values represent useful proxy values for recommending and modulating the intensity of ambulatory behavior and/or as measurement thresholds for processing accelerometer data.

**Trial registration:**

Clinicaltrials.gov (NCT02650258).

**Electronic supplementary material:**

The online version of this article (10.1186/s12966-019-0769-6) contains supplementary material, which is available to authorized users.

## Introduction

Objective monitoring of physical activity has quickly advanced since the millennium with the increasing and widespread availability of a variety of research- and consumer-grade wearable technologies. It is evident, however, that despite the diversity of design, most technologies capable of monitoring the wearer’s physical activity offer step counting as one of the detectable metrics. Step counting has been embraced by researchers [1], clinicians [2], and consumers [3] as an intuitively simple approach to communicating physical activity volume, expressed typically as steps/day. More recently, it has become recognized that the time-stamped sampling nature of accelerometry-based physical activity monitors also uniquely lends itself to the minute-by-minute study of ambulatory behavior in terms of cadence (steps/min) enacted in free-living contexts [4].

Cadence and stride length combine to determine speed of ambulation. Cadence is the principal strategy for increasing over-ground ambulatory speed, at least up to a self-selected preferred speed [5]. At least six studies [6–11] have proposed a cadence of 100 steps/min as a reasonable heuristic threshold (evidence-based, practical, rounded value) associated with absolutely-defined moderate intensity (3 metabolic equivalents, METs; 1 MET = 3.5 mL/kg/min of O_2_ consumption), which is the minimal level of intensity recommended in public health physical activity guidelines [12, 13]. Further investigation is required to confirm this heuristic threshold in a purposefully sex-and-age structured sample, and also to consider other cadence thresholds across a broader spectrum of MET-determined levels of intensity up to and including vigorous intensity (i.e., 3, 4, 5, and 6 METs). This information is critical to providing a minimally processed and translatable objectively monitored metric with established intensity thresholds across the lifespan.

The primary aim of the CADENCE-Adults study was to identify heuristic cadence thresholds associated with increasing intensity during walking. It expands on CADENCE-Kids, a preliminary study of cadence and intensity in 6–20 year olds [14]. This initial manuscript in the adult data series reports sex-and-age balanced data collected from 21 to 40 year olds (comparable to previously published samples). It represents the first installment in a planned series arising from the CADENCE-Adults study which upon completion will establish heuristic cadence-intensity thresholds for walking across the adult lifespan of 21–85 years old.

## Methods

### Study design and regulatory information

CADENCE-Adults is a laboratory-based cross-sectional study, conducted in the Physical Activity and Health Laboratory, Department of Kinesiology, University of Massachusetts Amherst. The study protocol was approved by the University of Massachusetts Amherst Institutional Review Board. Informed consent was obtained from all participants prior to enrolment and data collection. The study was also registered with Clinicaltrials.gov (NCT02650258). Study recruitment for this cohort (21–40 year olds) began in December 2015, and the data was collected between January and October, 2016.

### Participants and sample size calculation

Based on unpublished pilot testing, we determined that a minimum sample size of 8 participants per 5-year age category between 21 and 40 years (21–25, 26–30, 31–35, 36–40 years of age) was required to estimate the sample mean value of cadence associated with 3 METs to within ±10 steps/min with 95% confidence. To ensure a balanced sex-and-age distribution across the targeted age range, and to accommodate for the possibility of attrition and/or incomplete data, 10 men and 10 women for each 5-year age-group category were recruited, for a total of 80 participants. This strategic recruitment plan minimizes important sources of bias (i.e., sex and age) and improves the generalizability of the findings. Because the study’s intended focus was on ambulatory activity, potential participants who used wheelchairs or had other impairments that prevented normal ambulation were excluded. Additional exclusion criteria were Stage 2 hypertension (systolic blood pressure ≥ 160 mmHg or diastolic blood pressure ≥ 100 mmHg), current tobacco use, hospitalization for mental illness within the previous 5 years, body mass index (BMI) < 18.5 kg/m^2^ or > 40 kg/m^2^, cardiovascular disease or stroke, conditions or medications that could affect heart rate response to exercise, pacemakers or other implanted medical devices, and pregnancy. The 2013 edition of the American College of Sports Medicine Resources for The Health Fitness Specialist as well as the American Heart Association’s risk stratification recommendations presented in the same American College of Sports Medicine resource [12] were used to establish risk stratification. Following this screening process, low risk individuals were enrolled in the study and prepared for metabolic testing. Moderate risk individuals were also enrolled in the study and prepared for metabolic testing, but with blood pressure monitored throughout the procedures. High risk individuals received a physical examination including a resting electrocardiogram test prior to testing.

### Measures

*Race/ethnicity* was self-reported and captured for descriptive purposes.

*Standing Height* was measured to the nearest 0.1 cm (without shoes) using a wall-mounted stadiometer (ShorrBoard® Infant/Child/Adult Portable Height-Length Measuring Board; Weigh and Measure LLC, Olney, Maryland, USA). Measurements were repeated and a third measurement was taken if the first two differed by > 0.3 cm. The two closest measurements were averaged.

*Leg Length* was derived from seated height measured to the nearest 0.1 cm with a stadiometer. The participant was seated on a bench with their legs hanging freely and hands set on knees. Again, measurements were repeated and a third measurement was taken if the first two differed by > 0.3 cm. The two closest measurements were averaged. Seated height reflects the difference between the floor-to-crown measure and the static height of the bench. Leg length was then calculated by subtracting the seated height from standing height.

*Weight* was assessed (without socks or shoes) using a scale (DC-430 U; Tanita Corporation, Tokyo, Japan). Weight was measured to the nearest 0.1 kg. Again, up to three measurements were taken if the first two measurements differed by > 0.5 kg. The two closest measurements were averaged.

*Body Mass Index (BMI)* was calculated by dividing body weight by height squared (kg/m^2^). BMI-determined weight categories were: normal or healthy weight (18.5–24.9 kg/m^2^), overweight (25.0–29.9 kg/m^2^), or obese (≥30 kg/m^2^) [15].

*Waist Circumference* was measured using a non-elastic anthropometric measuring tape to the nearest 0.1 cm. The measurement was taken at the narrowest point between the iliac crest and lower costal border. Two measurements were taken, with a third required only if the first two differed by > 0.5 cm. The two closest measurements were averaged.

*Physical Activity Intensity* (oxygen consumption; VO_2_ mL/kg/min) was measured using a validated portable indirect calorimeter (Jaeger Oxycon Mobile; CareFusion BD Germany 234 GmbH, Höchberg, Germany) [16]. Heart rate was measured with a chest strap (Polar T31 Coded Transmitter; Polar Kempele, Finland). Self-reported rating of perceived exertion (RPE) was queried of each participant during the last minute of each bout using the Borg scale [17].

*Cadence (steps/min)* was directly observed (hand-tallied) and counted as steps accumulated during each bout. A video camera was also aimed at the participant’s feet to provide a redundant recording. Total steps tallied in each bout were divided by the duration of the bout (tallied steps/5-min) to calculate cadence in steps/min.

### Treadmill testing procedures

Participants began by sitting in a chair positioned on the treadmill for at least 5 min to establish baseline oxygen consumption values. The chair was then removed and participants were asked to walk for up to twelve 5-min bouts at a 0% grade. The test increased in 0.5 mph increments from 0.5 mph (13.4 m/min) to a maximum of 6.0 mph (160.9 m/min), with a 2-min standing rest between bouts (for a complete list of miles/h, km/h, and m/min conversions, see Additional file 1). Treadmill testing was terminated following completion of the bout when the participant: 1) naturally selected to run instead of walk; 2) exceeded 75% of age predicted heart rate maximum [0.75 x (220-age)]; 3) indicated an RPE > 13; or 4) chose to stop the protocol. Additionally, research staff could terminate the protocol if concerned for the participant’s safety.

### Data processing and aggregation

Metabolic data were imported in 5-s epochs, and step data were entered, into MATLAB (The MathWorks, Natick, MA) for all analyses using custom scripts. Mean VO_2_ values during minutes 2:45–3:45 and 3:45–4:45 of each 5-min trial were averaged. Metabolic equivalents (METs) were obtained by dividing the mass-specific VO_2_ (mL/kg/min) by 3.5 [18]. Moderate intensity ambulation was defined as ≥3.0 and < 6.0 METs, while vigorous intensity ambulation was defined as ≥6.0 METs [19].

### Analytic sample

Data from four of the 80 enrolled participants were not included for analysis due to equipment malfunction. Specifically, their oxygen consumption data did not increase during treadmill testing, remaining relatively similar to resting levels. Thus, a total of 76 participants were included in this analysis. The analytical data set comprised 612 treadmill walking bouts. All walking bouts were included in the analytical sample, irrespective of whether the individual did or did not reach an absolutely-defined moderate or vigorous intensity, since these bouts remained important for the statistical modelling procedures used. In addition, bout data for individuals who reached one or more of the termination criteria (see Treadmill Testing Procedures above) were included, provided they completed (walked) for the full 5-min bout. Running bouts (only achieved by 15 participants) were excluded from this analysis as the findings reported herein expressly focused on walking cadences. The final analytic dataset and corresponding data dictionary can be viewed in Additional files 2 and 3, respectively, formatted in accordance with the preceding CADENCE-Kids study [14] for compatibility.

### Statistical analyses

All statistical analyses were performed using R (version 3.0.2, R Foundation for Statistical Computing, Vienna, Austria). Statistical significance was set at α = 0.05. Descriptive statistics (mean and standard deviation for continuous variables, counts and percentages for categorical variables) were calculated for participant characteristics.

#### Preliminary analyses

The initial intent was to fit a linear or curvilinear model to the data representing the relationship between cadence and VO_2_, with cadence and METs as the independent and dependent variables, respectively. However, upon visual inspection of the data, a nonlinear relationship was observed between cadence and intensity that could not be appropriately described using a curvilinear fit. Moreover, the curvilinear model exhibited an ecologically invalid description of the data. That is, this model displayed increasing intensities at decreasing cadences below ~ 50 steps/minute and approached a vertical asymptote at the higher cadences. Thus, a segmented regression or ‘hockey stick’ model with both random and fixed coefficients was implemented. This model assigned two distinct (i.e., different slopes and intercepts) linear portions to the data. The value for the segment break point was chosen based on an iterative process to determine the point that minimized mean square error. A random coefficients model was selected to account for the repeated measurements of each participant. To compare the fit of the segmented regression to the curvilinear model, we performed a k = 5 cross-validation analysis with 10 repetitions and ascertained the root mean square error (RMSE).

#### Primary analyses

A fixed and random coefficients model was applied to the data to quantify the cadence-intensity relationship. Because participant repeated measures were accounted for in the model, marginal R^2^ values were obtained and reported as a description of model fit. Using the model’s regression equation and ± 95% prediction intervals (PIs), we solved for incremental cadence thresholds corresponding to 3, 4, 5 and 6 METs. Sensitivity, specificity, positive predictive value (PPV; i.e., the probability that an individual walking at a given cadence threshold would achieve the desired intensity level) and negative predictive value (NPV) were then quantified for each regression-identified threshold. In addition, Receiver Operating Characteristic (ROC) curve analysis was performed and optimal cadence thresholds corresponding to 3, 4, 5 and 6 METs were identified using Youden’s index [20]. Sensitivity, specificity, PPV, NPV and area under the curve (AUC) of these cadence thresholds are reported. Confidence intervals (99%) for optimal thresholds, and area under the curve (AUC) were obtained using the bootstrap with 20,000 replicates. Based on previously published standards [21], AUC values were interpreted as excellent (≥ 0.90), good (0.80–0.89), fair (0.70–0.79), and poor (< 0.70).

#### Secondary analyses

Leg length and sex are two participant characteristics that can affect cadence [5], and thus may affect the cadence-intensity relationship. Therefore, both of these variables were included as additional factors in separate segmented regression models. A k = 5 cross-validation analysis with 10 repetitions was performed to assess whether models that include either of these additional factors improved overall prediction (measured via RMSE).

### Heuristic cadence threshold determinations

Heuristic cadence thresholds were set as rounded multiples of 5 steps/min from the more precise MET-associated estimates identified from the segmented regression model and ROC curves. In the event that the two analytical approaches produced estimates that differed, we considered the trade-off in sensitivity, specificity, PPV and NPV for each candidate threshold to ultimately select a single heuristic threshold corresponding to 3, 4, 5, and 6 METs. While being mindful of the potential tradeoff in sensitivity and specificity of the thresholds, we leaned towards selecting values to produce a harmonious and incremental set of cadence thresholds that would have greater utility for researchers, clinicians and practitioners to flexibly recommend, modulate, and/or analyze ambulatory intensity. The set heuristic thresholds were then separately evaluated using ROC curve analysis to determine the sensitivity, specificity, PPV, NPV and AUC for identifying increasing levels of intensity. In addition, the classification accuracy of these heuristic thresholds (i.e., counts and percentages of correctly classified bouts as true positives and true negatives and falsely classified bouts as false positives and false negatives) were calculated.

## Results

### Sample characteristics

Descriptive characteristics of the 76 adults included in this analytic sample are reported in Table 1. As per our strategic recruitment plan, the sample was evenly distributed by sex and age. The sample was 30.4 ± 5.8 years of age, with a BMI of 24.8 ± 3.4 kg/m^2^, and predominately Caucasian (63.2%). In addition, we have also included a data summary table (Table 2) comprising the sample sizes, cadences, VO_2_, and MET values for each treadmill speed.

**Table 1:** Descriptive characteristics of the analyzed sample

Variable	Men (*n* = 38)		Women (*n* = 38)		Total (*N* = 76)	
	Mean	SD	Mean	SD	Mean	SD
Age (years)	30.3	6.3	30.6	5.3	30.4	5.8
Weight (kg)	80.6	13.5	65.0	9.9	72.8	14.1
Height (cm)	177.1	7.1	164.3	6.5	170.7	9.3
Leg length (cm)	83.5	4.7	76.0	4.2	79.7	5.8
BMI (kg/m^2^)	25.6	3.6	24.1	3.1	24.8	3.4
	n	%	n	%	n	%
BMI classifications						
Normal weight	19	50.0	25	65.8	44	57.9
Overweight	16	42.1	12	31.6	28	36.8
Obese	3	7.9	1	2.6	4	5.3

BMI categories: normal or healthy weight (18.5–24.9 kg/m^2^), overweight (25.0–29.9 kg/m^2^), obese (≥30 kg/m^2^) [15]

**Table 2:** Sample sizes, cadences, VO_2_, and METs for treadmill bouts

Treadmill Speed (mph)	*n*	Cadence (steps/min)	Min-Max	VO_2_ (mL/kg/min)	Min - Max	METs	Min-Max
0.5	76	45.4 ± 12.4	28–101	7.4 ± 1.1	5.0–11.0	2.1 ± 0.3	1.4–3.1
1.0	76	67.8 ± 9.1	53–105	8.3 ± 1.2	5.3–11.3	2.4 ± 0.4	1.5–3.2
1.5	76	83.8 ± 8.0	72–110	9.2 ± 1.2	6.3–11.9	2.6 ± 0.4	1.8–3.4
2.0	76	96.1 ± 6.5	85–115	10.2 ± 1.2	7.0–12.5	2.9 ± 0.3	2.0–3.6
2.5	75	105.8 ± 6.1	93–121	11.8 ± 1.2	7.9–14.1	3.4 ± 0.4	2.3–4.0
3.0	74	113.6 ± 6.1	101–127	14.2 ± 1.7	9.2–17.6	4.0 ± 0.5	2.6–5.0
3.5	70	121.5 ± 7.0	108–147	17.3 ± 2.2	10.7–24.9	5.0 ± 0.6	3.1–7.1
4.0	62	129.0 ± 7.6	115–161	21.5 ± 2.7	13.7–27.6	6.2 ± 0.8	3.9–7.9
4.5	34	139.9 ± 9.3	124–158	27.3 ± 3.8	16.0–33.7	7.8 ± 1.1	4.6–9.6
5.0	7	146.4 ± 7.3	135–158	30.6 ± 5.8	20.3–36.1	8.8 ± 1.7	5.8–10.3
5.5	1	152.0	NA	29.7	NA	8.5	NA

### Segmented regression with random coefficients model

**Fig. 1:**
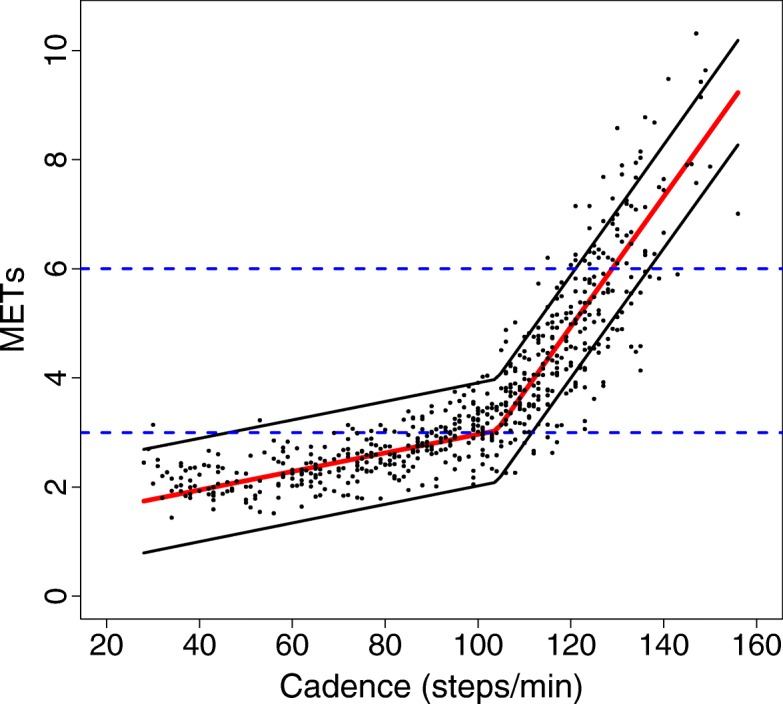
Relationship between cadence and METs using a segmented regression model. Red line is the mean METs value at each corresponding cadence value, and black lines are the 95% Prediction Intervals. Blue horizontal dotted lines represent moderate (3 METs) and vigorous intensity (6 METs), respectively

As indicated above, the data displayed two distinct linear trends, with the second displaying a much steeper relationship than the first (model break-point = 104 steps/min, marginal R^2^ = 0.84, Fig. 1). Adding leg length or sex to separate models did not change the break point. The segmented regression exhibited considerably less RMSE (0.68 ± 0.10) compared to the curvilinear model (2.74 ± 0.48). Moreover, the addition of leg length or sex to the segmented regression model did not improve the RMSE (0.68 ± 0.10 and 0.69 ± 0.10 when adding leg length and sex, respectively). Optimal cadence thresholds for increasing intensity levels (identified using the regression equation) are reported in Table 3. Briefly, the optimal cadence threshold for 3 METs was 102.2 steps/min and 129.1 steps/min for 6 METs.

**Table 3:** Cadence thresholds (steps/min) for moderate and vigorous intensity based on regression and ROC curve analyses

Intensity METs	Measure	Regression thresholds		ROC thresholds		Heuristic thresholds
		Value	95% PI	Value	99% CI	Value
3	Threshold (steps/min)	102.2	45.9–111.2	95.5	91.5–105.5	100
	Se	80.5	–	91.3	–	86.0
	Sp	92.5	–	86.2	–	89.6
	PPV	93.3	–	89.5	–	91.4
	NPV	78.7	–	88.5	–	83.3
	AUC	–	–	0.95	0.94–0.98	–
4	Threshold (steps/min)	112.5	103.5–120.2	112.5	105.5–113.5	110
	Se	89.0	–	89.0	–	93.5
	Sp	93.0	–	93.0	–	88.3
	PPV	86.0	–	86.0	–	79.6
	NPV	94.6	–	94.6	–	96.6
	AUC	–	–	0.97	0.96–0.99	–
5	Threshold (steps/min)	120.2	112.5–127.8	116.5	114.5–120.5	120
	Se	85.7	–	95.0	–	88.2
	Sp	93.7	–	89.2	–	92.7
	PPV	76.7	–	68.1	–	74.5
	NPV	96.5	–	98.7	–	97.0
	AUC	–	–	0.97	0.96–0.99	–
6	Threshold (steps/min)	129.1	121.4–136.8	119.5	119.5–125.5	130
	Se	64.1	–	98.4	–	64.1
	Sp	96.9	–	85.8	–	96.9
	PPV	70.7	–	44.7	–	70.7
	NPV	95.8	–	99.8	–	95.8
	AUC	–	–	0.97	0.95–0.99	–

95% Prediction Intervals (PI). 99% Confidence Intervals (CI). *AUC* area under the curve, *PPV* Positive Predictive Value, *NPV* Negative Predictive Value, *Se* sensitivity, *Sp* specificity

### Receiver operating characteristic analyses

Optimal cadence thresholds for increasing levels of intensity (identified using ROC analyses) are presented in Table 3. In summary, cadences of 96 steps/min and 120 steps/min corresponded to absolutely-defined moderate and vigorous intensities, respectively. Sensitivity and specificity values for these moderate and vigorous cadence thresholds were all > 85%, and AUC values were > 0.95, indicating excellent overall accuracy.

### Heuristic thresholds

**Fig. 2:**
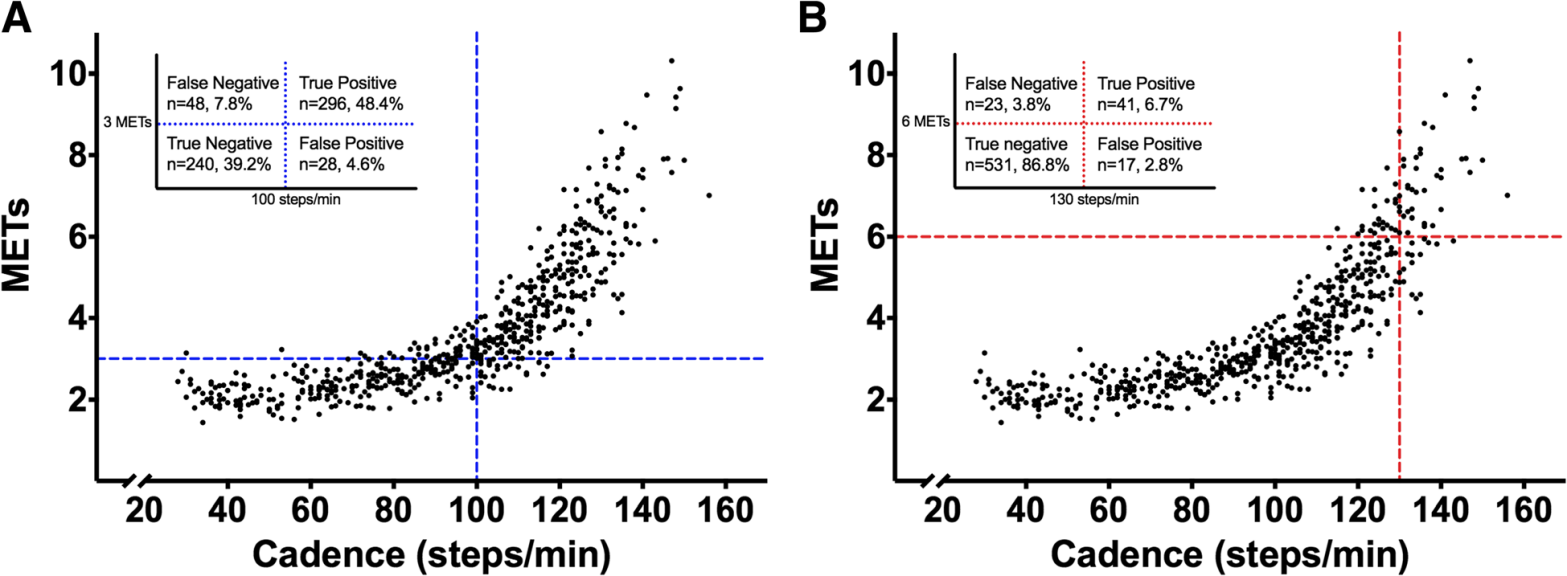
Classification accuracy of heuristic cadence thresholds and MET intensities. **a** 100 steps/min and 3 METs, **b**) 130 steps/min and 6 METs). The figure inserts display the values for true positives, false positives, true negatives and false negatives that were used to determine classification accuracy (sensitivity, specificity, positive predictive, and negative predictive values)

Heuristic cadence thresholds consistent with all incremental MET values anchored by 3 and 6 METs are presented in Table 3. To reiterate, these cadence thresholds were selected based on a compromise between the regression and ROC curve-based thresholds (rounded to the nearest 5 steps/min). Where the regression and ROC approaches yielded different candidate heuristic thresholds, we considered the trade-off in sensitivity, specificity, PPV and NPV for both candidate thresholds to ultimately select heuristic thresholds corresponding to 3, 4, 5, and 6 METs. As noted above, we deliberately leaned towards selecting values to create a harmonious and incremental set of thresholds consistent with the intentional use of these heuristic thresholds to recommend, modulate or quantify ambulatory behavior from a public health perspective. Ultimately, a heuristic cadence threshold of 100 steps/min emerged for 3 METs and 130 steps/min for 6 METs. Further, each 10 steps/min increase was roughly associated with an increase in intensity of 1 MET. Specifically, 4 METs was associated with 110 steps/min and 5 METs with 120 steps/min. Sensitivity and specificity for these heuristic thresholds closely resembled the regression- and ROC curve-based optimal thresholds. Classification accuracy determined using counts and percentages of correctly classified bouts (true positives, true negatives) and falsely classified bouts (false positives and false negatives) using moderate and vigorous intensity cadence thresholds are reported in Fig. 2. In total, 87.6% of bouts were correctly classified using the 100 steps/min threshold (Fig. 2; true positives plus true negatives), and 93.5% of bouts were correctly classified using the 130 steps/min threshold. The PPV for achieving a moderate intensity at 100 steps/min was 91.4%, and the PPV for achieving a vigorous intensity at 130 steps/min was 70.7%.

## Discussion

The CADENCE-Adults study is the first calibration study to employ a sex-and-age balanced sampling approach to establish heuristic cadence thresholds associated with increasing absolutely-defined intensity during walking. Using two distinct analytical methods, we confirm that 100 steps/min is a reasonable heuristic threshold associated with absolutely-defined moderate intensity (i.e., 3 METs) ambulation in 21–40 year olds. We also provide further evidence for additional cadence thresholds associated with incremental MET-defined intensity up to and including 130 steps/min as a heuristic threshold associated with 6 METs. These additional heuristic values are important indices useful for public health purposes to guide 1) generalized cadence-based walking recommendations and 2) analysis and interpretation of minimally processed ambulatory data obtained from contemporary wearable technologies.

Heuristic values are evidence-based, practical, rounded numbers that are grounded in evidence, but may not be necessarily precise. They serve as useful and easy to recall mental short cuts, quickly conveying generalized or broadly representative information to guide decisions. A simple daily-use example of a heuristic value is the estimated time it would take to drive between two cities. Other common public health-related examples of heuristic values include “eat 5 fruits and vegetables per day”, “be active 30 min/day”, and “limit time spent watching TV to 2 h/day”. It bears emphasizing here that heuristic values, while evidence-based and thus appropriate for public health purposes, are by definition not individualized.

We first proposed the heuristic value of 100 steps/min as a proxy indicator of moderate intensity in 2005, based on a linear regression model of treadmill walking [9]. A number of other studies [6–8, 10, 11] subsequently confirmed this heuristic value, despite acknowledging evidence of a tolerable range of inter-individual variation. Notably, these studies have been generally small, included predominantly younger samples, did not always employ a direct observation criterion standard of step counting, and employed various analytical approaches. This initial article focused on 21–40 year olds from the CADENCE-Adults study represents the largest sex-and-age structured sample to date employing a direct observation standard and using both regression and ROC analysis to inform evidence-based but generalized heuristic cadence values associated with absolutely-defined moderate and vigorous intensity. The 100 steps/min threshold for absolutely-defined moderate intensity continues to be confirmed for this age group. The stability of this heuristic across the adult lifespan up to 85 years of age will continue to be tested as part of the CADENCE-Adults study as data collection is completed.

To date, there have been three studies that have reported values congruent with a heuristic value of 130 steps/min associated with 6 METs (i.e., absolutely-defined vigorous intensity) in ostensibly healthy adults [6, 9, 22]. Herein, the optimal absolutely-defined vigorous intensity cadence thresholds were 129 and 120 steps/min, identified using regression and ROC analyses, respectively. Both algorithms are commonly accepted means of determining associations between independent and dependent variables and establishing thresholds. However, both analyses have different assumptions, and therefore different limitations. Regression models may be overly influenced by outliers, while ROC curves are organized in a rank-order fashion. By incorporating both methods, we provide more robust support for the heuristic thresholds reported herein. With that said, setting a lower threshold increases sensitivity, but reduces the specificity and PPV; the opposite is true for higher thresholds. Considering these trade-offs, we settled on a final heuristic threshold of 130 steps/min for absolutely-defined vigorous intensity.

The heuristic thresholds of 100 and 130 steps/min demonstrated good-to-excellent classification of absolutely-defined moderate and vigorous intensity ambulation, with an overall accuracy (true positive and true negative rates of > 85%). Moreover, for individuals walking ≥100 steps/min (~ 53.6–67.1 m/min or ~ 2.0–2.5 mph; Table 2), the probability (PPV) of achieving an absolutely-defined moderate intensity was 91.4%. For 130 steps/min (107.3 m/min or ~ 4.0 mph), the probability (PPV) of achieving an absolutely-defined vigorous intensity was 70.7%. This value is less than ideal and may be influenced by the lower number of participants (*n* = 49) who achieved 6 METs. However, this number still reflects 65% of the participant pool, and the associated NPV of 95.8% conversely suggested a very high probability that individuals walking at < 130 steps/min were at an intensity < 6 METs. Overall, this evidence supports the use of 100 and 130 steps/min, corresponding to absolutely-defined moderate and vigorous intensity ambulatory activity, respectively, as direct translations of public health recommendations for the minimum desired ambulatory intensity required to achieve health and fitness improvements [12, 13].

In the current study, we employed an absolutely-defined measure of intensity (i.e., METs), as opposed to a relatively-defined measure of intensity (e.g., %VO_2_Reserve, % Heart Rate Maximum [HR_max_] or Heart Rate Reserve [HRR]). This approach is consistent with previous studies that have determined accelerometer activity count cut points related to absolutely-defined moderate and vigorous intensities [23–25], and also with U.S. Federal physical activity guidelines [13, 26] and the American College of Sports Medicine position stand [27] that express their physical activity recommendations (intended for public health applications) using METs (e.g., 500–1000 MET-min/week). However, the use of absolute intensity may not be ideal for all adults, especially individuals who are older or have low fitness levels, whereby an indicator of absolute intensity represents a higher percentage of maximal capacity (relative to a younger or fitter adult) [27]. Few studies have examined the cadence-intensity relationship using relatively-defined measures of intensity, which may be more suitable for clinical or other types of individualized applications. For example, Serrano et al., [28] and Slaght et al. [29] reported cadence thresholds of 115 ± 10 and 114 ± 11 steps/min, respectively, associated with 40% of VO_2reserve_. In addition, Pillay et al., [30] found that 122 ± 37 steps/min corresponded to 60% of HR_max_, whereas O’Brien et al., [11] reported that ~ 120–125 steps/min corresponded to 40% MET_max_, dependent on the modelling technique and the covariates included in the model (e.g., height, leg length). The differences observed between these cadence thresholds (employing different relative indicators of intensity) and those reported herein (absolutely-defined) reflect the inconsistencies between the implemented intensity definitions. Unlike absolute intensity measures, for which there is consensus in the literature regarding what constitutes a moderate or vigorous intensity (3 and 6 METs respectively) [26, 27], there appears to be less consensus regarding relatively-defined intensity [31]. Using a single example of %HR_max_, moderate intensity has been defined as 64–76% HR_max_ [27], 55–69% HR_max_ [32], and 60% HR_max_ [30]. While there are strengths to using a relative intensity approach, especially for clinical and other types of individualized applications, there are also weaknesses, such as the need for a maximal fitness test to establish relative moderate and vigorous intensity levels based on individualized maximal/peak VO_2_ or HR values. Although it is possible to use equations to estimate %HR_max_ or HRR [33–37], such estimates are based on assumptions that may introduce an additional source of error. Indeed, there is no universally accepted HR-based equation with a minimal and acceptable (< 3 bpm) level of error [38]. Furthermore, some equations may be age (e.g., Åstrand [37]) or sex specific (e.g., Gulati et al., [34]), so care must be taken when applying these equations to various populations. Collectively, this makes such indicators of relative intensity less practical for public health applications including translations of physical activity guidelines as they are currently expressed [13, 26]. In summary, we believe our approach to using absolutely-defined intensity is reasonable and defensible given the consistency with previous studies and with public health guidelines. Still, we anticipate future research will be able to delve into the utility and limitations of individualizing cadence-based exercise prescriptions for clinical and more individualized applications (e.g., personal training).

An innovation of this study includes proffering a more comprehensive set of incremental cadence-intensity thresholds, including optimal and heuristic cadence thresholds for the intermediary values of 4 and 5 METs. Notably, with each increasing intensity level, the precision estimates (prediction intervals for regression; confidence intervals for ROC curve) tended to narrow, suggesting greater confidence that individuals walking at higher cadence thresholds will indeed achieve the desired higher intensity level. Based on the values presented herein, it is reasonable to conclude that, starting from 100 steps/min, each 10 steps/min increase is roughly associated with an increase in intensity of 1 MET, confirming the findings of a small preliminary study conducted in 2005 [9]. Notably, based on the regression and ROC optimal thresholds (both 112.5 steps/min) corresponding to 4 METs, we may have selected either 110 or 115 steps/min. However, considering our definition of a heuristic threshold (not only evidence-based, but also practically useful) and the complete set of cadence-intensity thresholds being put forth herein, we settled on 110 steps/min. In numerical terms, this was associated with a decrease in the PPV (8.3%) and increase in the NPV (4.6%) for this intensity level. Notably, these cadence thresholds, including that associated with 6 METs, are all achievable within the range of walking cadences for healthy adults; the walk to run transition occurs at ~ 140 steps/min [39]. Moreover, in the current study we deliberately excluded the bouts where 15 participants transitioned to running, so the evidence presented herein solely arises from walking cadence. With walking being the most commonly reported and widely accessible form of physical activity [40], this intentional focus greatly improves the utility of this set of cadence-intensity thresholds for application in the general population.

Regarding precision of regression predictions, we chose to report prediction intervals (PIs). While confidence intervals are more commonly reported, PIs are more appropriate for repeated measures dataset regressions, as they account for not only the uncertainty of the actual population mean, but also the overall spread of the data. For this reason, PIs appear wider in distribution compared to confidence intervals. Cadence PIs for 3 METs were seemingly large (45.9–111.2 steps/min). It is important to note that we intentionally included all walking bouts (e.g., starting at 0.5 mph) in order to incorporate a maximal range of ambulatory speeds. However, extremely slow speeds (e.g., 0.5 and 1.0 mph) may be considered non-ecological, as young healthy adults do not typically walk at these slow speeds and we observed our own participants struggling to find a comfortably paced execution of these speeds. In a different study, even when instructed to walk ‘rather slowly,’ healthy young adults (19–39 years old) chose to walk at a pace of 2.1 ± 0.4 mph [41]. When excluding the two slowest walking speeds employed herein, the mean cadence associated with 3 METs slightly decreases (96.4 steps/min), but more importantly the PIs tighten considerably (72–114 steps/min).

While the purpose of this analysis was to establish heuristic cadence-intensity thresholds in 21–40 years olds using group aggregate data, we acknowledge that inter-individual variability exists and that any heuristic threshold will have limited precision in terms of applicability to any single individual. While we accounted for the potential influence of both leg length and sex in the overall model fit across all participants, these additional variables did not change the model prediction (RMSE 0.68 ± 0.10 and 0.69 ± 0.10, respectively, compared to 0.68 ± 0.10 for the base model). Furthermore, the addition of leg length only marginally improved the model fit (R^2^ = 0.85; compared to the basic model, R^2^ = 0.84). Notably, the regression model including leg length predicted only a 0.58 MET difference at a given cadence between participants with the longest versus shortest leg length (95.5 cm vs. 65.7 cm, respectively). Similarly, when BMI was added to the regression model, the model fit did not change (R^2^ = 0.84), and there was only a 0.57 MET difference in predictions for participants with the highest and lowest BMI (36.9 vs 19.4 kg/m^2^, respectively). Given the limited change in model accuracy when adding these additional factors, we considered it reasonable to only include cadence in the final model. We acknowledge that any remaining variance in intensity at a given cadence may be better explained by other factors. In addition, we did not measure VO_2peak_ or VO_2max_ in this study, and as such are unable to make any conclusions regarding fitness and its impact on our study outcomes, or provide cadence thresholds corresponding to relative intensity measures. It bears repeating here, however, that the goal of establishing cadence-based thresholds corresponding to absolutely-defined intensity levels is to provide clear guidelines with little or no additional individual information required. Finally, we also acknowledge that cadence is specific to bipedal locomotor movements and further that these thresholds are most applicable to walking behaviors that are characteristically rhythmic, purposeful, continuous, and advancing forward through space.

Despite these limitations, cadence thresholds associated with absolutely-defined moderate and vigorous ambulatory intensity can serve as important heuristic values in efforts to measure and modulate adult walking behaviors, thus extending the potential utility of contemporary wearable technologies that offer step counting and cadence tracking features. One clear application of these cadence thresholds is for implementation in walking interventions. In our recent systematic review [42], we identified a limited number (*n* = 9) of intervention studies that had used a cadence-based goals to modulate walking behavior, or used cadence thresholds to quantify physical activity intensity from accelerometers and wearable device data. Based on the small number of studies and the observed associated high risk of bias, we concluded that it was premature to synthesize their findings. Rigorously designed walking intervention studies that utilize these cadence thresholds to convey and evaluate ambulatory behavior are required to elucidate the associated health benefits (e.g., improvements in aerobic fitness, blood pressure and glucose levels, body composition). In addition, future research should also explore ways to individualize cadence-based intensity prescriptions (e.g., using indicators of relative intensity) similar to Slaght et al., [29] and to modulate intensity in predictable ways (e.g., manipulating cadence using rhythmic auditory cueing [music or metronome]).

## Conclusions

In summary, 100 steps/min and 130 steps/min are acceptable heuristic cadence thresholds associated with absolutely-defined moderate and vigorous intensity walking, respectively, in 21–40 year olds. Each 10 steps/min increase is roughly associated with an increase in intensity of 1 MET such that 4 METs is associated with 110 steps/min and 5 METs with 120 steps/min. Future reports from the CADENCE-Adults study will either confirm these values or establish age-appropriate heuristic thresholds for walking across the adult lifespan of 21–85 years of age. Additional research is needed to gauge the utility and limitations of individualized cadence-based prescriptions potentially linked to indicators of relative intensity.

## Additional files

Additional file 1: Table displaying miles/h and km/h conversions. (PDF 46 kb)

Additional file 2: Table displaying final analytical data set. (XLS 161 kb)

Additional file 3: Table displaying a data dictionary for Additional file 2. (XLS 28 kb)

## Abbreviations

AUC: Area under the curve
BMI: Body mass index
METs: Metabolic equivalents
mph: Miles per hour
NPV: Negative predictive value
PI: Prediction interval
PPV: Positive predictive value
ROC: Receiver operating characteristic
RPE: Rating of perceived exertion
Se: Sensitivity
Sp: Specificity
VO_2_: Oxygen consumption
