# Supplementary material for: Walking cadence (steps/min) and intensity in 21–40 year olds: CADENCE-adults
Source: Int J Behav Nutr Phys Act. 2019 Jan 17;16:8. doi: 10.1186/s12966-019-0769-6 (PMC6337834; doi:10.1186/s12966-019-0769-6)
Supplement: Supplementary file 1 — Table displaying miles/h and km/h conversions. (PDF 46 kb) [file 12966_2019_769_MOESM1_ESM.pdf]

Cadence Adults Additional File 1: Treadmill bouts speed conversions			
**Bout**	**m/min**	**km/h**	**miles/h**
1	13.4	0.8	0.5
2	26.8	1.6	1.0
3	40.2	2.4	1.5
4	53.6	3.2	2.0
5	67.1	4.0	2.5
6	80.5	4.8	3.0
7	93.9	5.6	3.5
8	107.3	6.4	4.0
9	120.7	7.2	4.5
10	134.1	8.0	5.0
11	147.5	8.9	5.5
12	160.9	9.7	6.0
